# A role for sperm in regulation of egg-laying in the Nematode *C. elegans*

**DOI:** 10.1186/1471-213X-7-41

**Published:** 2007-05-01

**Authors:** Marie McGovern, Ling Yu, Mary Kosinski, David Greenstein, Cathy Savage-Dunn

**Affiliations:** 1 Department of Biology, Queens College, and The Graduate School and University Center, CUNY, Flushing, NY 11367, USA; 2 Department of Genetics, Cell Biology, and Development, University of Minnesota, Minneapolis, MN 55455, USA; 3 Department of Biology, New York University, New York, NY 10003, USA

## Abstract

**Background:**

In insects and in mammals, male sperm and seminal fluid provide signaling factors that influence various aspects of female physiology and behavior to promote reproductive success and to compete with other males. It is less apparent how important such signaling is in the context of a self-fertile hermaphrodite species. We have addressed this question in the nematode *Caenorhabditis elegans*, which can reproduce either by hermaphrodite self-fertilization or by male-hermaphrodite mating.

**Results:**

We have studied the egg-laying defective mutant, *egl-32*, and found that the cellular basis of the *egl-32 *egg-laying phenotype is likely a defect in sperm. First, the time of *egl-32 *action coincides with the timing of spermatogenesis in the hermaphrodite. Second, *egl-32 *interacts with genes expressed in sperm. Third, mating experiments have revealed that wild-type sperm can rescue the egg-laying defect of *egl-32 *mutant animals. Most importantly, introduction of mutant *egl-32 *sperm into wild-type hermaphrodites or females is sufficient to induce an egg-laying defective phenotype.

**Conclusion:**

Previous work has revealed that *C. elegans *sperm release factors that stimulate oocyte maturation and ovulation. Here we describe evidence that sperm also promote egg laying, the release of embryos from the uterus.

## Background

Egg laying in *C. elegans *is a carefully monitored process. Genetic studies of egg-laying defective (Egl) mutants have revealed significant insight into the neuronal, developmental, and environmental control of egg laying behavior [1]. Egg laying is biphasic, alternating between an active phase when eggs are laid, and an inactive phase [2]. The hormone serotonin is necessary to initiate the active phase of egg laying. Serotonin is produced by the hermaphrodite specific neurons (HSNs) [3]. Hermaphrodites integrate many internal and external cues in deciding whether or not to enter the active phase. Wild-type hermaphrodites under laboratory conditions release embryos from the uterus at a similar stage in their development. Retaining fertilized eggs for too long endangers the mother's life, as larvae that hatch internally will consume and destroy the mother. There are circumstances, however, such as in the absence of food, where egg retention may be desirable, as this increases the odds that the progeny will be laid in a favorable environment. In addition, when eggs are retained, they eventually hatch internally and devour the hermaphrodite's body before escaping the cuticle, allowing maternal resources to be sacrificed to the progeny. Accordingly, in the absence of food, worms enter the active phase less frequently, and animals begin to bloat with eggs [4]. Neurotransmitters that inhibit egg laying are also known. For example, the endocrine uv1 cells produce tyramine which acts to inhibit egg laying [5]. This inhibitory pathway may allow egg-laying behavior to respond to the internal cue of the presence of fertilized eggs in the uterus [6]. In studies of the egg-laying defective mutant *egl-32*, we have now uncovered another internal cue, the presence of sperm, which stimulates egg laying.

*C. elegans *hermaphrodites produce sperm during the fourth larval (L4) stage [7]. The sperm are amoeboid not flagellated, and they are stored in the spermathecae. When the worms undergo their final molt to become adults they switch to producing exclusively oocytes and will do so for the remainder of their lives. As the oocytes mature they are ovulated and pass though the spermathecae where they come in contact with sperm. The oocytes are fertilized, pass into the uterus, and are eventually laid. Unlike in humans, almost all of the sperm in the hermaphrodite are used to fertilize oocytes [8]. The number of sperm limits the number of potential offspring a self-fertilizing hermaphrodite is capable of producing, while matings with males will increase the number of progeny [9]. Males produce exclusively sperm beginning in the L4 stage and throughout their adult lives. If a male is allowed to mate with a hermaphrodite his sperm will crawl into the spermatheca where they will be preferentially used to fertilize the oocytes[8]. Previous work has revealed that *C. elegans *sperm release factors that stimulate oocyte maturation and ovulation [10,11]. Here we describe evidence that sperm also promote egg laying, the release of embryos from the uterus.

## Results

### Characterization of the egg-laying defects of egl-32 mutants

**Figure 1:**
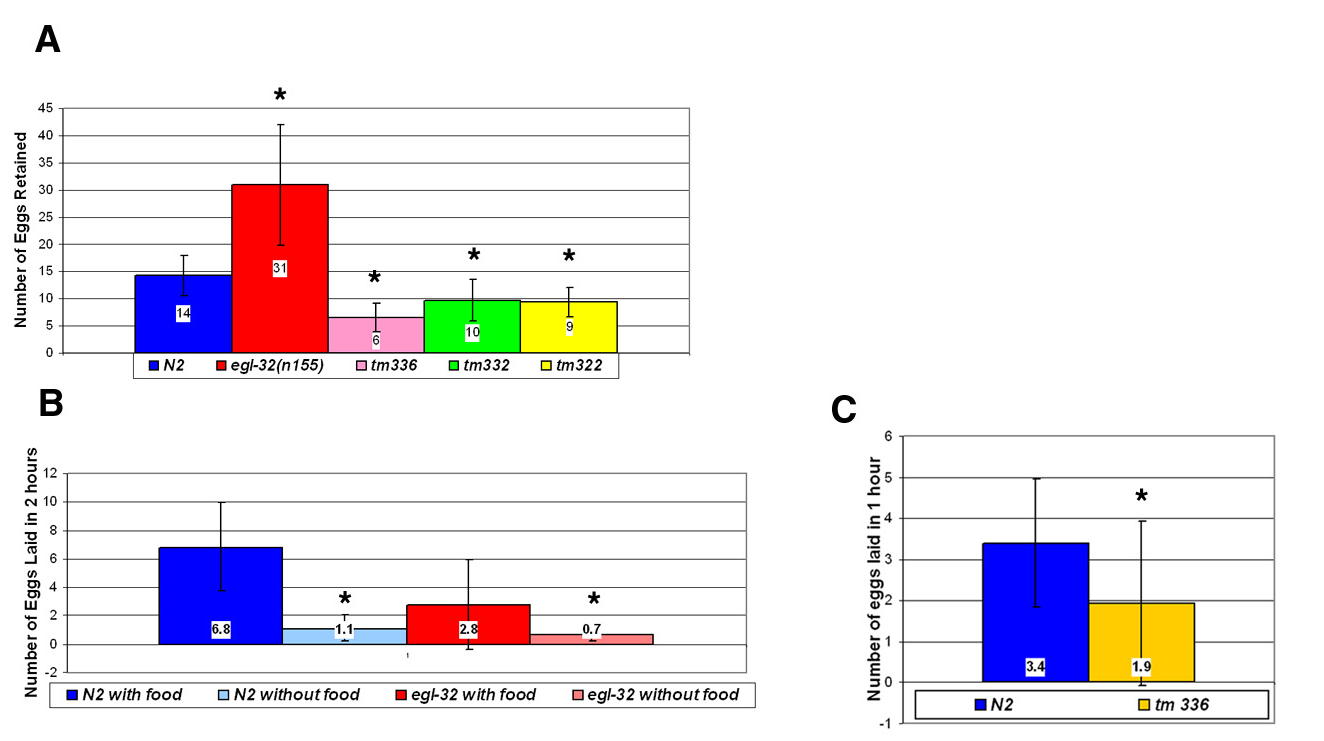
Egg Retention – (a) *egl-32(n155) *animals retain about twice as many eggs as wild type animals. Animals containing a deletion in T08G11.2 (*tm33*6) and animals containing deletions in two of T08G11.2's homologues (*tm332 *and *tm322*) retain far fewer eggs than wild-type animals. 36 wild-type N2 animals, 36 *egl-32(n155) *animals, 35 *tm336 *animals, 67 *tm332 *animals and 144 *tm322 *animals were used in this assay. * Statistically significant difference. Student t-test value < 0.05. Error bars in this and all figures show standard deviation. (b) – Mutant *egl-32 *animals lay about half as many eggs as wild-type animals. However, mutant *egl-32 *animals do respond normally to food cues. They begin to lay even fewer eggs in the absence of food. 26 N2 animals in the presence of food, 39 N2 animals in the absence of food, 26 *egl-32 *animals in the presence of food, and 38 *egl-32 *animals in the absence of food were used in this assay. * Statistically significant difference. Student t-test value < 0.05. (c) – T08G11.2 deletion mutants (*tm336*) lay significantly fewer eggs than wild-type animals. However, they retain late stage embryos. * Statistically significant difference. Student t-test value < 0.05.

In large-scale genetic screens for egg-laying defective (Egl) mutants, a single temperature-sensitive allele of the gene *egl-32*, *n155*, was isolated [12]. Pharmacological tests designed to characterize gene activity relative to the HSNs did not identify the cellular or anatomical basis for the *egl-32 *egg-laying defect. Here we present evidence that the cellular basis of the *egl-32 *egg-laying phenotype is a defect in sperm. We used two assays to quantify egg laying, an egg retention and an egg laying assay (Methods). In the egg retention assay, mutant *egl-32 *animals retain about twice as many eggs as wild-type animals (Figure 1a). In the egg-laying assay, mutant *egl-32 *animals lay about half as many eggs per time period as wild-type animals (Figure 1b).

**Figure 2:**
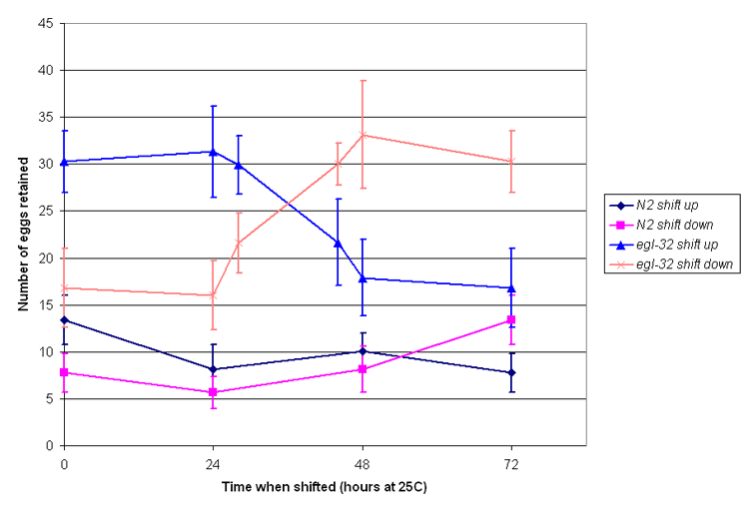
Temperature Shift Assay – Temperature shift assay reveals that 24 to 48 hours is the critical period for *egl-32 *activity. This corresponds to the L4 stage of development. Time 0 is the time at which parental adult hermaphrodites were picked. Progeny were scored after 72 hours at 25°C or equivalent developmental time. 10–30 animals were used at each time point.

Because food cues play an important role in egg laying, we were interested in determining whether *egl-32 *mutant animals have a normal response to food. In the absence of food wild-type animals lay fewer eggs. This is also true of *egl-32 *animals (Figure 1b) suggesting that the defect in *egl-32 *is not at the level of chemosensation or sensory processing of food cues. The one existing allele of *egl-32*, *n155*, displays a temperature-sensitive egg-laying defect at the nonpermissive temperature of 25°C. We performed a temperature shift assay to determine the critical period for *egl-32 *activity. The temperature sensitive period is between 24 and 48 hours after egg collection at 25°C, which corresponds to the L4 stage of development (Figure 2). This result was interesting because egg laying does not occur at this stage. However, this is the time during which vulval morphogenesis occurs and the HSNs innervate their targets. Moreover, it is the one and only time during which hermaphrodites produce sperm [7].

### egl-32 interacts with T08G11.2

**Figure 3:**
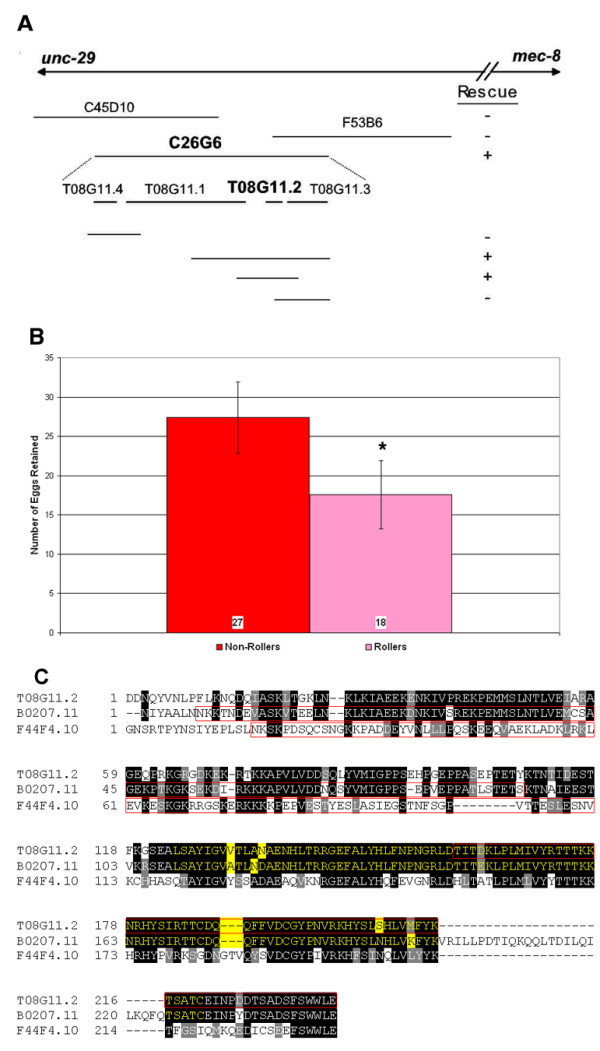
Rescue Experiments – (a) Cosmids from the region thought to be associated with *egl-32 *were injected into mutant *egl-32(n155) *animals. One cosmid from this interval, C26G6, which overlaps the sequenced cosmid T08G11, rescued the *egl-32 *phenotype. Among several subclones of this cosmid, one containing two predicted ORFs T08G11.2 and T08G11.3, but not one containing only T08G11.3, also rescued *egl-32*. (b) – Injection of the subclone along with *rol-6 *rescues the egg-laying defect of *egl-32*. * Statistically significant difference. Student t-test value < 0.05. (c) – Alignment of T08G11.2 with B0207.11 and F44F4.10. Identical amino acid residues are highlighted in black, similar amino acids in gray. The SH2-like domain is highlighted in yellow. Sequences deleted in deletion alleles are outlined in red. For the internal deletions in B0207.11 and F44F4.10 we have not determined whether an in-frame product is created.

In transformation experiments, we identified a cosmid, C26G6, which could rescue the *egl-32 *phenotype. Subclones of this cosmid containing the predicted open reading frame (ORF) T08G11.2 can also rescue *egl-*32 (Figures 3a &3b). Several lines of evidence, however, suggest that *egl-32 *does not encode T08G11.2, but rather that they are interacting loci. First, when we sequenced T08G11.2 from *egl-32(n155) *mutants we could find no change to the genome. We extended our sequencing of this region from the previous ORF to the next ORF and still could not find a change to the genome. Second, SNP mapping was performed (data not shown), that confirmed that *egl-32 *is separable from T08G11.2. Third, we performed a complementation test between *egl-32 *and a T08G11.2 deletion allele, *tm336*. The trans-heterozygotes *tm336*/*n155 *retained an average of 13 eggs, significantly fewer than *n155 *homozygotes (p = 1.74 × 10^-14^). Thus, *tm336 *complements *egl-32*, supporting the conclusion that they are separate but genetically interacting genes.

A BLAST search revealed that T08G11.2 has three homologues, B0207.11, F44F4.10 and F42G4.6 in *C. elegans *(Figure 3c). T08G11.2 and its three homologues have all been identified as sperm enriched transcripts [13]. T08G11.2 and its closest homologue, B0207.11, have about a 10,000-fold enrichment in sperm. A similar fold enrichment is found in the sperm specific proteins (*ssp-11*, *ssp-16*, *ssp-19*, *ssq-1*, *ssq-2*, *sss-1 *and *sss-2*). T08G11.2 encodes a small novel protein of 282 amino acids. It contains an SH2-like domain. However, it lacks the critical arginine present in functional SH2 domains. To characterize further the biological roles of these sperm enriched proteins, we obtained deletion alleles from the *C. elegans *knockout consortium. *tm336 *contains a deletion in T08G11.2, *tm322 *in the ORF B0207.11, and *tm332 *in the ORF F44F4.10. These animals all retained far fewer eggs than wild-type animals (Figure 1a), suggesting the absence of an egg-laying defect. However, older embryos were often seen in mutant hermaphrodites, which would be consistent with an egg-laying defect. We therefore determined the egg-laying rate for *tm336*, the T08G11.2 mutant, to resolve this issue (Figure 1c). The data demonstrate that *tm336 *is, in fact, egg-laying defective based on rates of egg laying. The decreased egg retention is therefore not due to increased egg laying, but may reflect a reduced ovulation rate instead (see below).

### Mating experiments

We became interested in the possibility that sperm play an active role in egg laying when we discovered that the *egl-32 *interacting gene T08G11.2 and its three homologues are all highly expressed in sperm. This hypothesis is supported by the temperature shift assay, which revealed that the L4 stage of development, the stage at which hermaphrodites produce sperm, is the critical period for *egl-32 *activity (Figure 2). We investigated the possibility that sperm are playing an active role in egg laying by performing mating experiments. In all of these experiments, the mutation *him-5 *was included in the genetic background to facilitate recovery of males. Males were stained with a vital dye for a minimum of 2 hours and allowed to mate with stage-matched young adult hermaphrodites. Hermaphrodites were then examined for the presence or absence of fluorescent sperm as an indicator of the success or failure of mating. If the *egl-32 *egg-laying defect is due to a defect in the sperm, then we might expect that the introduction of wild-type sperm could correct this defect. Consistent with this hypothesis, the experiments revealed that the introduction of wild-type sperm, via mating, into *egl-32 *mutant animals rescued the egg-laying defect (Table 1). More strikingly, the introduction of mutant *egl-32 *sperm into wild-type animals was sufficient to induce an egg-laying defective phenotype (Table 1). The presence of males in itself did not lead to this change in egg-laying behavior, since control animals on the same plates without detectable stained sperm were not affected significantly. In addition, in control experiments with *egl-32;him-5 *hermaphrodites mated with *egl-32;him-5 *males, the hermaphrodites retained an average of 31 eggs. This increase in egg retention was not statistically significant, and demonstrates that mating *per se *does not rescue the egg-laying defect.

**Table 1:** Mating Experiments

Hermaphrodite	Male	Eggs retained	n
*egl-32;him-5*	not mated	22 ± 6	89
*egl-32;him-5*	*him-5 *(mating not detected)	19 ± 5	60
*egl-32;him-5*	*him-5*	13* ± 3	77
*egl-32;him-5*	*egl-32;him-5*	31 ± 8	39
*egl-32;him-5*	*spe-38;him-5*	24 ± 3	10
*egl-32*	*spe-9 *(mating not detected)	24 ± 4	20
*egl-32*	*spe-9;him-5*	24 ± 3	20
*him-5*	not mated	11 ± 3	71
*him-5*	*egl-32;him-5 *(mating not detected)	13 ± 4	80
*him-5*	*egl-32;him-5*	20* ± 8	38
*fog-2*	*him-5*	13 ± 4	29
*fog-2*	*egl-32;him-5*	18* ± 5	38
*fog-2*	*him-5 *(20°C)	7 ± 3	104
*fog-2*	*egl-32;him-5 *(20°C)	7 ± 3	84
*egl-33*	*him-5 *(mating not detected)	27 ± 10	59
*egl-33*	*him-5*	28 ± 10	29

*Statistically significant difference between mated and control animals, p < 0.05.

All experiments are performed at 25°C, unless otherwise indicated. Data ranges give standard deviation.

To determine the effect of *egl-32 *mutant sperm in the absence of endogenous hermaphroditic sperm we performed mating experiments using female *fog-2 *animals. These mutants make oocytes but not sperm, and so are not self-fertile [14]. Consistent with our previous mating experiments, *fog-2 *animals mated with *egl-32 *males retained significantly more eggs than *fog-2 *females mated with wild-type males (Table 1). To confirm that the egg-laying defect is due to the inactivation of *egl-32*, we repeated these experiments at 20°C, a permissive temperature. At this temperature, there is no difference between females mated with *egl-32 *and those mated with wild-type males, consistent with the conclusion that *egl-32 *provides the relevant activity. Finally, we determined that the introduction of sperm cannot rescue every egg-laying defect. We tested *egl-33 *mutant animals by introducing wild-type sperm into *egl-33 *hermaphrodites (Table 1). The animals containing stained sperm did not retain fewer eggs than those not mated with wild-type males.

**Figure 4:**
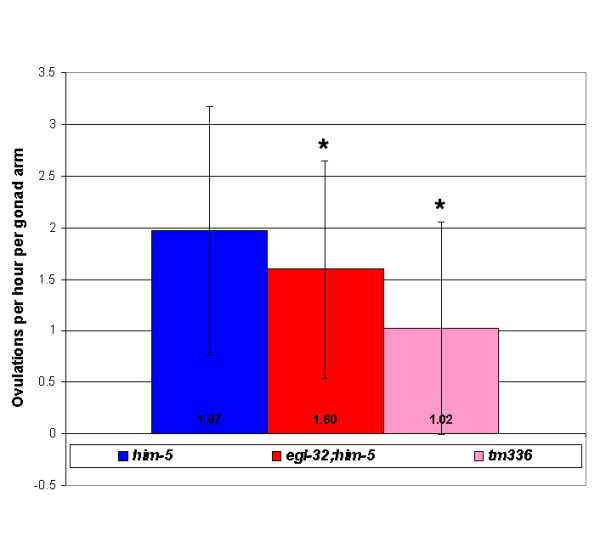
Ovulation Rate Assay – Mutant *egl-32;him-5 *animals ovulate at a slightly, but significantly, slower rate than *him-5 *animals. Animals containing a deletion in T08G11.2 (*tm336*) ovulate at a much slower rate than wild-type animals. 68 wild-type *him-5 *animals, 100 *egl-32;him-5 *animals and 53 *tm336 *animals were used in this assay. * Statistically significant difference. Student t-test value < 0.05.

These mating experiments established the existence of a sperm-derived signal, dependent on *egl-32 *activity, which promotes egg laying. The best characterized sperm signaling molecules are the Major Sperm Proteins (MSPs), which are necessary for oocyte maturation and ovulation [11]. In mutant female animals producing only oocytes, the oocytes will not mature and ovulation occurs at a very low rate. But, when MSPs are introduced, the oocytes are induced to mature and the sheath cells surrounding the ovary are stimulated to contract resulting in more frequent ovulations. Since a sperm-derived signal regulates ovulation rates, we tested whether our mutants have altered ovulation rates. We have found that the ovulation rate in *egl-32(n155) *mutant animals is slightly, but significantly reduced (Figure 4). The ovulation rate in *tm336 *mutants, in which the *egl-32 *interacting gene, T08G11.2 is deleted, is even more significantly reduced (Figure 4).

### Fertilization is required for egl-32 activity

In our mating experiments, we found that *egl-32 *mutant male sperm can suppress egg laying even in the presence of wild-type hermaphrodite sperm. The question arises as to why the hermaphrodite sperm are not sufficient to promote normal egg-laying rates. One possible explanation is that egg laying is coupled to fertilization. Since male sperm out-compete hermaphrodite sperm for fertilization of oocytes, in matings with *egl-32 *males the defective male sperm will be preferentially used for fertilization even in the presence of wild-type hermaphrodite sperm. We were therefore interested in determining if fertilization is required for the sperm-derived signal regulating egg laying. To determine if fertilization is required we mated *egl-32 *hermaphrodites with fertilization-defective males. Sperm from *spe-9 *and *spe-38 *mutants are structurally normal, can crawl into the spermatheca, but are incapable of binding to and fertilizing an oocyte [15,16]. In these mating experiments, we found that neither *spe-9 *nor *spe-38 *mutant sperm could rescue the *egl-32 *defect, unlike wild-type sperm (Table 1). This result eliminates the possibility that the rescuing activity is provided either by seminal fluid or by the physical act of mating rather than by some factor in the sperm. We propose instead that fertilization is coupled to the regulation of egg-laying rates.

### MSP localization in egg-laying defective mutants

**Figure 5:**
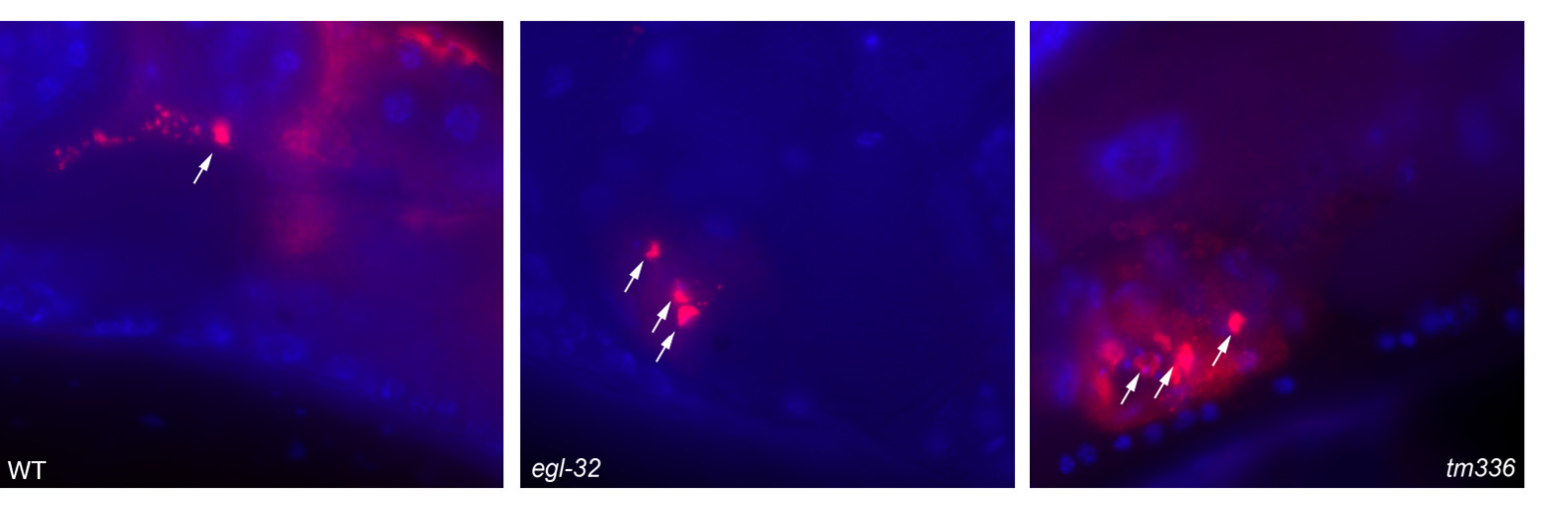
Detection of Extracellular MSP in the Uterus – Adult hermaphrodites were stained in whole-mount with monoclonal anti-MSP (red). DNA was detected with DAPI (blue). Bright red staining spermatozoa (arrows) are located near small red puncta and diffuse patches of staining, which likely correspond to extracellular MSP vesicles and free MSP, respectively [17]. While the patterns of sperm localization and MSP staining are slightly variable in the uterus, the staining observed in the *egl-32(n155) *and *tm336 *mutants was qualitatively within the normal range seen in the wild type. Sperm are approximately 4 μm in size.

Interestingly, *spe-9 *sperm are capable of signaling to promote meiotic maturation [15-17], but we find them insufficient to promote egg laying. Thus, these two sperm functions are separable genetically. Since MSPs are the active factor in promoting meiotic maturation, we asked whether a defect in MSP production or localization could also underlie the egg-laying defects of *egl-32(n155) *and *tm336 *mutants. MSP has previously been found to be present in the uterus, where it could theoretically be involved in regulating egg laying. MSP localization was visualized by immunohistochemistry (Figure 5). We found that in both mutant backgrounds, detectable levels of MSP are released and are present in the uterus, arguing against a role for MSP in regulating egg laying downstream of *egl-32 *and T08G11.2. Although we cannot exclude the existence of subtle differences in MSP localization or accessibility, or the existence of isoform-specific differences in these mutants, our results suggest that the egg-laying defects are not due to a loss of MSP signaling. This conclusion is consistent with the observation that regulation of meiotic maturation and of egg laying are genetically separable.

## Discussion

Mutant *egl-32 *hermaphrodites retain about twice as many eggs, lay about half as many eggs and ovulate at a slightly, but significantly slower rate than wild-type animals. The reduction in egg laying rate is not due to a defect in chemosensation or sensory processing of food cues as *egl-32 *animals respond normally to food cues. Several lines of evidence suggest that the defect in *egl-32 *is due to an abnormality of the sperm. First, the *egl-32 *gene interacts with T08G11.2, a gene that is highly expressed in sperm, to promote proper egg laying in *C. elegans*. Injection of T08G11.2 into *egl-32(n155) *mutant animals rescues their egg-laying defective phenotypes. Second, *egl-32(n155) *is a temperature-sensitive mutation. Temperature shift assays reveal that the L4 stage of development is the critical period for EGL-32 activity. This was an interesting finding because this is not when eggs are being laid. However, it is the one and only time that a hermaphrodite produces sperm. Third, mating experiments reveal that wild-type sperm introduced, via mating, into *egl-32 *animals rescues their egg-laying defective phenotype. This is not true of all egg-laying defective mutants, as the egg-laying defect of *egl-33 *could not be rescued via mating. Fourth, *egl-32 *mutant sperm are sufficient to confer the egg retention phenotype in both wild-type hermaphrodites and females. We have also determined that fertilization of oocytes is required to mediate *egl-32 *activity. Mutant *spe-9 *and *spe-38 *sperm, which are incapable of fertilizing oocytes, were unable to reduce the number of eggs retained by mutant *egl-32 *hermaphrodites when introduced via mating. Thus, we conclude that *egl-32 *activity is required for a sperm-derived signal that promotes egg laying and acts at or after fertilization. This signal is unlikely to be MSP, since MSP localization is not detectably altered in *egl-32*. Furthermore, the regulation of meiotic maturation/ovulation and egg laying are at least partially separable genetically as seen in *egl-32(n155) *mutants, which have significant defects in egg laying but only a slightly reduced ovulation rate (this study), and in *spe-9 *mutants, which are defective in promoting egg laying but not in promoting meiotic maturation [17].

A role for seminal fluid in regulating female oocyte maturation and ovulation, among other behaviors, has been well established in *Drosophila *[18]. In particular, the seminal fluid hormone Acp70A (sex peptide) stimulates oogenesis and Acp26Aa (ovulin) stimulates ovulation. Similarly, factors in mammalian seminal fluid, such as TGFβ and prostaglandin, promote changes in the female to facilitate embryo survival and implantation [19]. The rapid evolution of male seminal fluid proteins suggests a role in male-female sexual conflict, in which the male benefits most by increasing the quantity of offspring, while the female benefits more by selecting high quality mates [20,21]. In *C. elegans*, although competition between hermaphrodite and male sperm occurs [8] it might be expected that the hermaphrodite's sperm would play a limited role in regulating its own reproductive behaviors due to the lack of sexual conflict. This, apparently, is not the case. Instead, hermaphrodite sperm actively signal to promote oocyte maturation and ovulation via the MSPs [11] and, as shown here, to promote egg laying via an *egl-32*-dependent activity. Sperm signaling in the hermaphrodite may exist to ensure prudent investment of resources. In the absence of sperm, ovulation would be an energetically wasteful behavior. Similarly, a sperm-derived signal may exist to coordinate the rate of fertilization to the rate of egg laying. As long as sperm are present the rate of egg laying is close to the rate of ovulation resulting in a fairly constant number of eggs present in the uterus. Older, wild-type worms normally bloat with eggs. These older hermaphrodites may become defective in egg laying because they no longer contain the internal cue present in sperm to promote egg laying. Conversely, egg laying in young adults is inhibited until the uterus becomes sufficiently filled with eggs, possibly by an inhibitory effect of the uv1 cells that is blocked when mechanical deformation by eggs occurs [6]. It is possible that the sperm signal modifies or coordinates with the uterine-occupancy signal to effect the appropriate rate of egg laying throughout the nematode's life history.

## Conclusion

Our data demonstrate the existence of a novel sperm-derived signal that regulates rates of egg laying in the nematode *C. elegans*. This signal is unlikely to be dependent on the known sperm signaling molecule MSP. The novel signal requires sperm fertilization of oocytes to function.

## Methods

### *C. elegans *stocks and genetics

The following worm stocks used in this study were obtained from the Caenorhabditis Genetics Center (CGC): *egl-32(n155) *I, *spe-9(hc88) *I, *him-5(e1490) *V, *fog-2(q71) *V. The following deletion alleles were obtained from Dr. Shohei Mitani, National Bioresource Project for the nematode, Tokyo Women's Medical University School of Medicine, Japan: *tm336 *I, *tm322 *I, *tm332 *II. In addition to phenotypes reported here, *tm322*, the deletion allele of B0207.11, has an Unc phenotype that cosegregates with the deletion. The *spe-38 *I; *him-5 *V animals were obtained from Andrew Singson. We generated *egl-32;him-5 *and *spe-9;him-5 *by standard techniques. Worms were raised at 25°C, unless otherwise noted, and grown on EZ worm plates (E. Lambie, personal communication; Per liter: 550 mg Tris-Cl, 240 mg Tris-OH, 3.1 g Bacto Peptone, 8 mg cholesterol, 2.0 g NaCl, 200 mg streptomycin sulfate, 20 g agar).

### Egg-laying assays

L4 larvae were placed on plates with food and allowed to mature at 25°C overnight to become young adults. The young adults were move to individual plates that either contained or lacked food. The worms were allowed to lay eggs at 25°C for 2 hours. The number of eggs (or progeny, as sometimes was the case with the egg-laying defective animals) present on the plate after 2 hours was then counted.

### Egg retention assay

L4 larvae were placed on plates with food and allowed to mature at 25°C overnight into young adults. The young adult worms were then placed on an agar pad on a glass slide in a drop of 10 mM sodium azide. The worms were then viewed under a 40 × objective lens (Zeiss Axioskop). The number of eggs retained in the uterus was counted.

### Ovulation rate assay

L4 larvae were placed on plates with food and allowed to mature at 25°C overnight into young adults. Worms were then placed in a small drop of OP50 on an agar pad. The number of eggs/oocytes contained in the uterus and spermathecae were counted. The worms were then transferred to individual plates containing food and allowed to lay eggs at 25°C for 5 hours. The worms were then again placed on a slide with an agar pad in a small drop of OP50 and the eggs retained were again counted. The number of eggs present on the plate was also counted. The ovulation rate was determined by taking the number of eggs retained originally and subtracting it from the number of eggs retained after 5 hours plus the number of eggs laid during the 5 hours. The average number of eggs/oocytes ovulated in 1 hour and per gonad arm was then calculated.

### Temperature shift assay

Eggs were placed on a plate and allowed to hatch at either 15°C or 25°C. At various times after hatching the worms where shifted to a higher or lower temperature [22]; the worms that hatched at 25°C were shifted down to 15°C; the worms hatched at 15°C were shifted up to 25°C. The number of eggs retained in the young adults was then counted as above. 10 – 30 animals were scored at each time point. Two hours of development at 15°C was considered equivalent to one hour of development at 25°C.

### Rescue by injection

The following clones and subclones were injected, along with *rol-6 *as a marker gene, in to the gonad of *egl-32(n155) *animals: C45D10, C26G6, F53B6, H14D01, F32H2, W06D4, F36F2, W10D5, ZK858, H07C08, H32K16, F25H5, C45G3, H07C08 and subclones of C26G6. We screened for rolling animals that were no longer egg-laying defective.

### Mating experiments

All animals used in mating experiments included the *him-5 *mutation in the genetic background. The hermaphrodites, or female *fog-2 *animals, used in the mating experiments were selected as L4 larvae were allowed to mature overnight at 25°C. The males used in the mating experiments were stained with SYTO-17 [23] as follows:

Worms were washed off plates with M9 solution and transferred to a 1.5 ml microcentrifuge tube. The worms were pelleted by microcentrifugation. The supernatant was removed. The dye was prepared by taking 3 μl of 5 mM stock solution into 1497 μl M9 to make a 10 μM solution. (occasionally a solution as concentrated as 100 μM was used. This was done only when the dye was old and appeared to be losing its ability to fluoresce strongly). 500 μl of dye was added to the worm pellet. 250 μl of dyed worms were transferred to two clean dry plates. The plates were wrapped in aluminum foil and places at 25°C for a minimum of 2 hours. To remove the dye solution the worms were pelleted in a fresh 1.5 ml microcentrifuge tube. The supernatant was removed. The worms were resuspended in a few drops of M9 and transferred to a fresh plate for recovery. Once the plates were dry stained males were picked to plates containing the young adult hermaphrodites and allowed to mate for a minimum of 2 hours. The hermaphrodites were then transferred to an agar pad on a microscope slide and placed in a drop of 10 mM sodium azide. The number of eggs retain in the uterus was then counted under a 40 × objective lens.

### MSP localization

Immunohistochemistry using anti-MSP monoclonal antibodies was performed as described previously [17].

## Authors' contributions

MM carried out the phenotypic studies, participated in the analysis of the study, and drafted the manuscript. LY carried out the temperature shift assays and the microinjection experiments. MK carried out the immunohistochemistry. DG participated in the design and analysis of the study. CSD conceived of the study, and participated in its design and coordination and helped to draft the manuscript. All authors read and approved the final manuscript.
